# High prevalence of soil-transmitted helminth infections in Myanmar schoolchildren

**DOI:** 10.1186/s40249-022-00952-6

**Published:** 2022-03-10

**Authors:** Eindra Aung, Kay Thwe Han, Catherine A. Gordon, Nyein Nyein Hlaing, Moe Moe Aye, Myo Win Htun, Khin Thet Wai, Su Mon Myat, Thida Lay Thwe, Aung Tun, Kinley Wangdi, Yuesheng Li, Gail M. Williams, Archie C. A. Clements, Susana Vaz Nery, Donald P. McManus, Darren J. Gray

**Affiliations:** 1 Department of Global Health, Research School of Population Health, Australian National University, Canberra, ACT Australia; 2 St Vincent’s Clinical School, University of New South Wales, Sydney, NSW Australia; 3 Parasitology Research Division, Department of Medical Research, Ministry of Health and Sports, Yangon, Myanmar; 4 Infectious Diseases Program, QIMR Berghofer Medical Research Institute, Brisbane, QLD Australia; 5 Department of Zoology, University of Yangon, Yangon, Myanmar; 6 Department of Public Health, Ministry of Health and Sports, Nay Pyi Taw, Myanmar; 7 Ministry of Health and Sports, Nay Pyi Taw, Myanmar; 8 Hunan Institute of Parasitic Diseases, World Health Organization Collaborating Centre for Research and Control On Schistosomiasis in Lake Region, Yueyang, China; 9 School of Public Health, University of Queensland, Brisbane, QLD Australia; 10 Faculty of Health Sciences, Curtin University, Perth, WA Australia; 11 The Kirby Institute, University of New South Wales, Sydney, NSW Australia

**Keywords:** *Ascaris lumbricoides*, Hookworm, *Ancylostoma*, *Necator americanus*, *Trichuris trichiura*, Soil-transmitted helminth, Myanmar, Real-time PCR, Kato Katz

## Abstract

**Background:**

Achieving the elimination of soil-transmitted helminth (STH) infections requires a sufficient understanding of the current epidemiological status of STH endemicity. We aimed to examine the status of STH in Myanmar – a country with the eighth highest STH prevalence in the world, 10 years after instigation of the national deworming programme.

**Methods:**

In August 2016 we screened for STH infections using Kato Katz (KK) microscopy and real-time PCR (qPCR) in schoolchildren from the Bago Region township of Phyu, a STH sentinel site in Myanmar. Ten schools were randomly selected, and one stool sample each from a total of 264 students was examined. Prevalence and intensity of infection were calculated for each STH.

**Results:**

High prevalence of STH was identified in the study area with 78.8% of the schoolchildren infected with at least one STH by qPCR, and 33.3% by KK. The most prevalent STH was *Trichuris trichiura*, diagnosed by both KK (26.1%) and qPCR (67.1%), followed by *Ascaris lumbricoides* (15.5% KK; 54.9% qPCR). No hookworm infections were identified by KK; however, the qPCR analysis showed a high prevalence of *Ancylostoma* sp. infection (29.6%) with few *Necator americanus* (1.1%) infections.

**Conclusions:**

Despite bi-annual deworming of schoolchildren in the fourth-grade and below, STH prevalence remains stubbornly high. These results informed the expansion of the Myanmar National STH control programme to include all school-aged children by the Ministry of Health and Sports in 2017, however further expansion to the whole community should be considered along with improving sanitation and hygiene measures. This would be augmented by rigorous monitoring and evaluation, including national prevalence surveys.

## Background

More than 1.5 billion people are currently infected with soil-transmitted helminths (STH) (*Ascaris lumbricoides*, *Trichuris trichiura* and the hookworms *Necator americanus* and *Ancylostoma* sp.) primarily in Asian, African, and Latin American countries [1, 2]. The STH are intestinal parasitic nematode worms that are some of the most common and disabling chronic human pathogens in the developing world [3]. STH are intimately associated with rural poverty, inadequate sanitation and poor hygiene, lack of clean water, and limited access to health care and preventive measures (e.g. health education)[3–5]. STH impact significantly on public health and may cause severe disability among the world’s poorest communities [1, 6, 7] – particularly among school-aged children (SAC), who, if chronically infected, may suffer from profound physical deficits, including anaemia and malnutrition, stunted growth, reduced fitness and cognitive delays [8–16].

Myanmar has the eighth highest prevalence of STH of any country worldwide, with an estimated 40.8 million people at risk, and requiring preventive chemotherapy, including nearly 13 million children living in endemic areas [16]. A Ministry of Health and Sports (MoHS) parasitological survey in 2002–2003 showed a 70% STH prevalence in 1,000 schoolchildren across four ecological areas for STH transmission (plains, hills, coast and delta) [17, 18]. Based on these results the MoHS introduced a National STH control programme in 2006 [19]. Forming part of the School and Youth Health Programme (SYHP), the STH control programme follows World Health Organization (WHO) guidelines, which are to provide bi-annual albendazole chemotherapy (400 mg single oral dose), usually in February and August, via targeted deworming in pre-SAC (aged 2–4 years) and SAC (aged 5–9 years) [19, 20]. In 2012, the MoHS repeated their 2002–2003 parasitological survey in 1,000 SAC, 7 years after the commencement of the national deworming programme. The survey results showed that whilst the STH prevalence had decreased to 21% overall, and 0% in hilly areas, the prevalence still remained high in the plains (30%), coastal (38%), and delta areas (15%) [19].

A cross-sectional survey of two Myanmar villages in 2015–2016 showed a high prevalence of 27.81% by Kato-Katz (KK) for human infection with at least one STH, peaking at 36% in the 5–14 year age group [21]. *T. trichiura* was the most prevalent STH, possibly due to the low efficacy of albendazole (ABZ) to treat this species compared with other STH [21, 22].

As STH control continues in Myanmar, ongoing rigorous monitoring and evaluation will be required to inform the MoHS on programme effectiveness and decision-making around up-scaling of control activities [23]. To achieve this, there is a requirement to utilize highly sensitive and specific methods for detecting infected individuals. The WHO recommends the KK thick smear technique for assessing both the prevalence and intensity of infection in helminth control programmes, and was used for all of the aforementioned surveys in Myanmar [24]. However, the KK technique lacks sensitivity, particularly with low-intensity infections, such as those occurring in areas with ongoing mass deworming programmes [25–28]. Molecular approaches are increasingly being used in monitoring and surveillance as they have higher sensitivity and can differentiate species (or genera) of hookworms, unlike KK [26, 29–34]. We previously showed that real-time PCR (qPCR) is considerably more sensitive than KK for STH detection in the Philippines [34, 35], a feature supported by a number of other recent studies in South East Asia [27, 32, 35–37].

Here we present the results of a parasitological survey we conducted among SAC in Phyu Township, Bago Region – a sentinel site for the Myanmar National STH control programme. Our aim was to determine the status of STH infection by both the KK and qPCR after 10 years of targeted deworming.

## Methods

### Study design

**Fig. 1:**
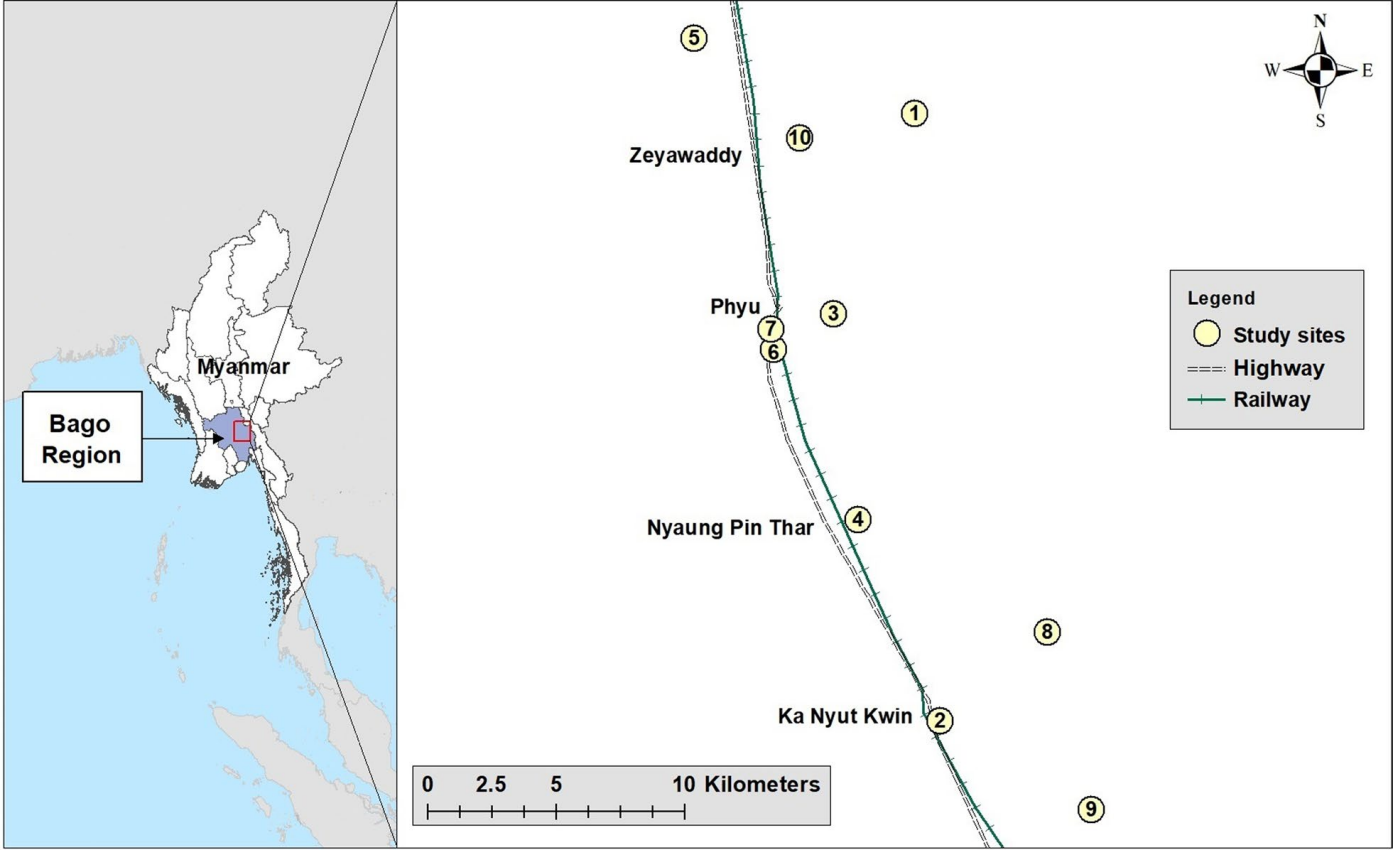
Map of Myanmar showing the study area in Bago Region (left panel); approximate locations of the ten participating schools in Phyu Township (right panel). (Source: Myanmar Information Management Unit and Google Maps)

A cross-sectional study was conducted in August, 2016 in Phyu Township, Bago Region, Myanmar (Fig. 1), to determine the prevalence of STH infections in SAC utilising KK and qPCR procedures.

### Ethical approval

Ethical approval was provided by the Protocol and Ethics Review Committee, the Department of Medical Research, Ministry of Health and Sports, Myanmar, (Approval Number: Ethics/DMR/2016/099), and the Science and Medical Delegated Ethics Review Committee, The Australian National University, Australia (Protocol: 2016/406).

### Study setting and population

The study was undertaken on grade five schoolchildren (aged 9–11 years) across ten randomly selected schools (5 urban; 5 rural) in Phyu Township – the sentinel site for the ‘plains’ ecological area in the previous Myanmar MoHS surveys [17, 19]. Phyu Township is situated 162 kms south of Nay Pyi Taw (the capital of Myanmar) and 185 kms north of Yangon (the former capital). There were 215 schools, enrolling a total of 5,120 fifth graders in Phyu Township for the 2016–2017 school year (June to February), with the number of fifth graders ranging from three to 154 per school.

The selection of the study participants was based on WHO guidelines [24], which recommends a target sample size of 50 per school in five randomly-selected schools in each ecologically homogenous area. Ten schools were randomly selected in this study as student numbers in most schools were less than 50. All grade five schoolchildren in the 10 schools (*n* = 363) were invited to participate to ensure enrolment of 250 children. De-worming was undertaken in primary schoolchildren (from kindergarten to grade four) in January and July in 2016 as part of the national STH control programme. The fifth graders recruited into this study were last treated in January 2016 before they completed grade four, and they were not treated in July 2016.

### Field and microscopy procedures

In each of the selected schools, the study team conducted a meeting with parents or guardians of the fifth graders to explain the study and obtain consent. Participating students were given a stool container and instructions on how to collect their stool at home and asked to provide one stool sample each. A questionnaire was also administered at the school to collect data on demographics; knowledge, attitudes and practices around intestinal worms and hygiene; and water and sanitation status. Samples were processed at the Phyu Township hospital within 2–3 h after collection and read the same day using triplicate KK thick smears (41.7 mg of stool/smear) [38]. For quality control, all of the slides were rechecked by the leader of the stool processing and examination team for the study. The team comprised four microbiologists (including the leader) and two research assistants from the Department of Medical Research in Myanmar and the Department of Zoology, the University of Yangon. Ten percent of the slides were examined by an independent microbiologist at a government hospital in Yangon for additional quality control.

Approximately 2–3 g of faeces collected from each participant was stored in 80% (v/v) ethanol for DNA extraction and qPCR analysis at the QIMR Berghofer Medical Research Institute (QIMRB), Brisbane, Australia.

### DNA extraction

DNA extraction was performed on all stool samples; 200 mg of stool were placed into a 2 ml screw cap tube and washed once with MilliQ water by adding 500 µl of MilliQ to each tube and centrifuging at maximum speed in a benchtop centrifuge (Eppendorf, Hamburg, Germany) for 3 min. Then the supernatant was removed and 500 µl of ROSE buffer [39] and 1 g of 0.5 mm silica/zirconia beads (Daintree Scientific Australia, St Helens, Australia) were added to each tube [32, 35]. The tubes were placed into a Precellys Homogeniser (Bertin Instruments, Paris, France) at 3306 × *g* for 30 s to fully homogenise the stool samples, a step crucial for rupturing *T. trichiura* eggs. The tubes were then placed in a heating block at 95 ℃ for 5 min, then centrifuged (Eppendorf, Hamburg, Germany) at maximum speed to pellet the sample. The supernatant was then added to the first well of a Maxwell 16 LEV Plant DNA Kit (Promega, Madison, USA) cartridge to which 200 µl of MilliQ water had already been added. The cartridge was then placed in a Maxwell 16 robot, along with elution tubes containing 50 µl of elution buffer, and plungers placed in well 8. On the Maxwell 16 robot the program for plant DNA was selected and automated extraction commenced. Once extraction was completed the elution tubes were removed. The Maxwell system uses magnetic beads for DNA extraction and some carryover of beads to the eluted DNA can occur. These can be removed by using a magnetic rack (Thermo Fisher Scientific, Waltham, USA) to attract the magnets to one side of the tube while the supernatant containing DNA can be removed and placed into a clean, labelled tube. DNA quality and quantity were verified using a Powerwave (BioTek, Winooski, USA).

### Multiplex qPCR

A multiplex qPCR was conducted using previously published primers and probes for *A. lumbricoides*, *T. trichiura*, *N. americanus*, and *Ancylostoma* sp. (Table 1) [32, 33, 35, 40]. Reactions in a total volume of 15 µl contained 8 µl of GoTaq (Promega), 3.2 µl of MilliQ water, and the appropriate concentrations of primers and probes for each species as shown in Table 1.

**Table 1:** PCR primers and probes used in this study

Organism	Target	References	Name	Fluorophore/Quencher	Final concentration (nmol/L)	Sequence (5' → 3')
*Ascaris lumbricoides*	ITS1	[33, 40]	Asc F	*FAM/Iowa Black*	60	GTAATAGCAGTCGGCGGTTTCTT
			Asc R		60	GCCCAACATGCCACCTATTC
			Asc P		100	TTGGCGGACAATTGCATGCGAT
*Ancylostoma* sp*.*	ITS2	[33, 40]	Anc F	*Cy5/Iowa Black*	200	GAATGACAGCAAACTCGTTGTTG
			Anc R		200	ATACTAGCCACTGCCGAAACGT
			Anc P		200	ATCGTTTACCGACTTTAG
*Necator americanus*	ITS2	[33, 40]	Nam F	*JOE NHS Ester/Iowa Black*	200	CTGTTTGTCGAACGGTACTTGC
			Nam R		200	ATAACAGCGTGCACATGTTGC
			Nam P		100	CTGTACTACGCATTGTATAC
*Trichuris trichiura*	ITS1	[37]	Trich F	*Cy5.5/Iowa black*	60	TCCGAACGGCGGATCA
			Trich R		60	CTCGAGTGTCACGTCGTCCTT
			Trich P		100	TTGGCTCGTAGGTCGTT

The qPCR was performed using a multiplex CFX384 (Biorad, Hercules, USA) under the following conditions: 2 min initialisation at 98 ℃, followed by 40 cycles at 98 °C for 20 s, 58 °C for 20 s, and 72 °C for 20 s, followed by a final extension at 72 °C for 5 min.

Genomic DNAs of *A. lumbricoides*, *T. trichiura*, *N. americanus*, and *Ancylostoma* sp*.*, extracted from adult worms or eggs, were used as positive controls. The control DNAs were combined to create aliquots comprising equal DNA quantities of the four species. A 1:10 dilution series (to 1:10^5^) of the DNA combination was used in each assay as a positive control and to allow comparisons of individual multiplex PCR assays. Two no template controls were also subjected to the multiplex PCR assay. All samples were analysed in triplicate.

Positive samples were determined using the average cycle threshold (Ct) for each triplicate sample. Standard deviation (*SD*) was calculated for the replicates. To be considered positive at least two replicates required a *SD* of < 1. Samples with positive Ct but > 1 *SD* between replicates were repeated. A Ct value < 35 was considered positive.

### Statistical analyses

Statistical analyses were carried out using Microsoft Excel 2016 (Microsoft, Albuquerque, New Mexico, USA), SPSS statistical software (IBM® SPSS® Statistics 23.0, Chicago, IL, USA), Venny 2.1 (BioinfoGP, Centro Nacional de Biotecnología, Spain), and GraphPad Prism 8 (GraphPad Software Inc., SanDiego, California, USA). A positive case of infection was determined by the presence of at least 1 egg on one KK slide or a qPCR Ct score of < 35. Descriptive statistics (frequency, percentages, means and standard deviations) were used to examine the main study variables (STH prevalence and intensity), the *t*-test was used to detect differences in means, and the chi-square test determined differences in proportions. Confidence intervals were calculated using a one sample *t*-test. Infection intensity was calculated using both arithmetic mean eggs per gram of faeces (AMEPG) and geometric mean eggs per gram of faeces (GMEPG) in infected individuals.

## Results

### Study participation

Overall participation rate was 76% (274/363) ranging from 50% to 100% among participating schools, with a total of 274 grade-five students partaking in the study and providing one stool sample each. All 274 stool samples were examined by KK; however, only 264 samples were examined by qPCR due to insufficient stool quantity in 10 samples. Participants that had both KK and qPCR (*n* = 264) results were included in the analyses presented here. The mean age of the participants was 11.0 ± 1.1 years (range: 9–16 years), with 57% (*n* = 150) aged 9–10 years, and 53% (*n* = 139) female. Overall, 65.5% of participants (*n* = 173) attended urban schools and the remainder (34.5%; *n* = 91) were from rural schools.

### STH infection prevalence

#### KK

STH infection determined by KK (*n* = 264) showed a prevalence of 33.3% (95% *CI*: 27.6–39.1) for at least one STH infection; 15.5% (95% *CI*: 11.1–19.9) (*n* = 41) for *A. lumbricoides*, and 26.1% (95% *CI*: 20.8–31.5) for *T. trichiura* (Table 2). No stool samples were positive for hookworm by KK.

**Table 2:** Prevalence and infection intensity of soil transmitted helminths determined by the Kato-Katz procedure, qPCR and the two methods combined

	KK				qPCR		KK and qPCR combined	
	*n*	Prevalence (95% *CI*)	AMEPG (95% *CI*)	GMEPG (95% *CI*)	*n*	Prevalence (95% *CI*)	*n*	Prevalence (95% *CI*)
*A. lumbricoides*	41	15.5% (11.1–19.9)	15,322.3 (7,566.5–23,078.2)	2,059.1 (892.5–4,750.1)	145	54.9% (48.9–61.0)	156	59.1% (53.1–65.1)
*T. trichiura*	69	26.1% (20.8–31.5)	382.5 (207.9–557.1)	123.9 (86.4–177.9)	177	67.1% (61.3–72.8)	197	74.6% (69.3–79.9)
*Ancylostoma* sp.		N/A	N/A	N/A	78	29.6% (24.0–35.1)		N/A
*N. americanus*		N/A	N/A	N/A	3	1.1% (0–2.4)		N/A
Hookworm	0	0%	N/A	N/A	81	30.7% (25.1–36.3)	81	30.7% (25.1–36.3)
Any STH	88	33.3% (27.6–39.1)	N/A	N/A	208	78.8% (73.8–83.8)	222	84.1% (79.7–88.5)
All 3 STH	0	0%	N/A	N/A	63	23.9% (18.7–29.0)	64	24.2% (19.0–29.5)
Any 2 STH	22	8.3% (5.0–11.7)	N/A	N/A	69	26.1% (20.8–31.5)	84	31.8% (26.2–37.5)
*A. lumbricoides* and T. trichiura*	22	8.3% (5.0–11.7)	N/A	N/A	118	44.7% (38.7–50.7)	134	50.8% (44.7–56.8)
*A. lumbricoides* and hookworm*	0	0%	N/A	N/A	65	24.6% (19.4–29.9)	65	24.6% (19.4–29.9)
*T. trichiura* and hookworm*	0	0%	N/A	N/A	75	28.4% (22.9–33.9)	77	29.2% (23.7–34.7)

*KK* Kato-Katz method, *n* Number of positive cases, *95% CI* 95% confidence interval, *AMEPG* Arithmetic mean of eggs per gram of faeces in infected children, *GMEPG* Geometric mean of eggs per gram of faeces in infected children, *Any STH* Infection with any of the three parasites (*A. lumbricoides*, *T. trichiura*, or hookworm), *All 3 STH* Infection with all three parasites, *Any 2 STH* Infection with any two of the three parasites, *N/A* Not applicable; *The number includes children infected with all three STH

#### qPCR

Analysis by qPCR (*n* = 264) found a prevalence of 78.8% (95% *CI*: 73.8–83.8) for at least one STH infection; 54.9% (95% *CI*: 48.9–61.0) for *A. lumbricoides,* 67.1% (95% *CI*: 61.3–72.8) for *T. trichiura*, and 30.7% (95% *CI*: 25.1–36.3) for hookworm [*Ancylostoma* sp*.* 29.6% (95% *CI*: 24.0–35.1); *N. americanus* 1.1% (95% *CI*: 0–2.4)] (Table 2).

#### qPCR vs KK

Combining the results from the KK and qPCR analysis indicated a prevalence of 84.1% (95% *CI*: 79.7–88.5) for any STH infection; 59.1% (95% *CI*: 53.1–65.1) for *A. lumbricoides*, and 74.6% (95% *CI*: 69.3–79.9) for *T. trichiura* (Table 2). As indicated above, no hookworm-positive individuals were identified by KK. When compared with the KK, the qPCR analysis identified approximately 3.5 times (145/41) the number of *A. lumbricoides* infections and 2.5 (177/69) times the number of *T. trichiura* infections.

**Fig. 2:**
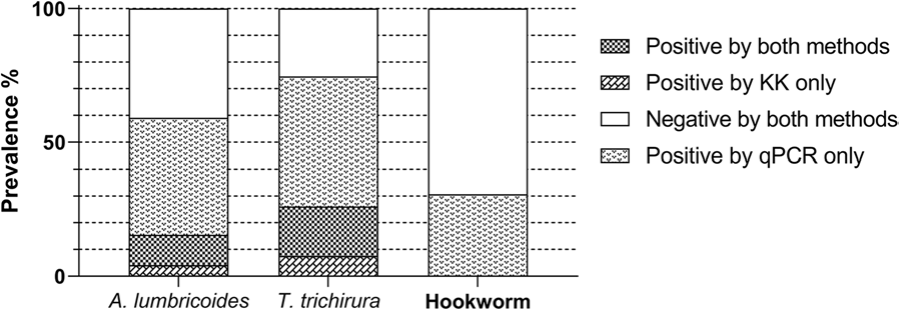
Prevalence of soil transmitted helminths determined by qPCR and Kato-Katz (KK) microscopy (*n* = 264)

A comparison between the qPCR and KK procedures showed that 11 samples positive for *A. lumbricoides* by the KK method were negative by qPCR, and 20 samples positive by KK for *T. trichiura* were negative by qPCR (Table 3 and Fig. 2). Conversely, 115 samples negative for *A. lumbricoides* by the KK method were found to be positive by qPCR, and 128 samples found to be negative for *T. trichiura* by KK were positive by qPCR.

**Table 3:** Comparison of the Kato-Katz and qPCR methods for diagnosis of *A. lumbricoides* and *T. trichiura*

	*A. lumbricoides*				*T. trichiura*		
	Positive qPCR	Negative qPCR	Total		Positive qPCR	Negative qPCR	Total
Positive KK	30	11	41		49	20	69
Negative KK	115	108	223		128	67	195
Total	145	119	264		177	87	264

#### Co-infections

**Fig. 3:**
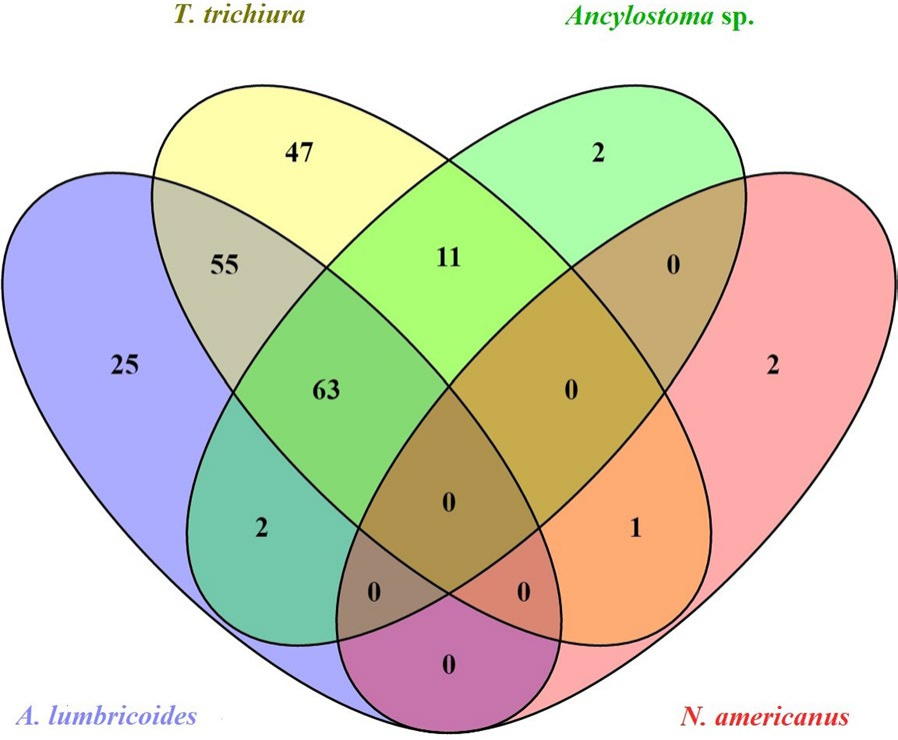
Venn diagram showing soil transmitted helminths co-infections in schoolchildren determined by qPCR (Total positive cases = 208)

The qPCR analysis indicated that 23.9% (95% *CI*: 18.7–29.0) of the schoolchildren were co-infected with all three STH (*A. lumbricoides*, *T. trichiura* and hookworm), and 26.1% (95% *CI*: 20.8–31.5) with any two STH (Table 2). Infections with *A. lumbricoides* and *T. trichiura* were found in 44.7% (95% *CI*: 38.7–50.7), *T. trichiura* and hookworm in 28.4% (95% *CI*: 22.9–33.9), and *A. lumbricoides* and hookworm in 24.6% (95% *CI*: 19.4–29.9) of schoolchildren (Table 2). The most common single infection was *T. trichiura* (*n* = 47) followed by *A. lumbricoides* (*n* = 25), while the most common co-infection was triple infection with *T. trichiura, A. lumbricoides*, and *Ancylostoma* sp. (*n* = 63) (Fig. 3).

#### STH prevalence and demographics

STH prevalence (using the combined KK and qPCR results) was stratified according to sex, age group and school location (Table 4). Males (34.4%; 95% *CI*: 27.0–41.8) had a significantly (*P* < 0.001) higher prevalence than females (15.1%; 95% *CI*: 8.1–22.1) for infections with all three STH, and a significantly (*P* = 0.002) higher prevalence of hookworm; 40.0% (95% *CI*: 32.0–48.0) for males compared with 22.3% (95% *CI:* 14.7–29.9) for females (Table 4). Children < 11 years were more likely to have any STH infection (88.7%; 95% *CI*: 82.8–94.5) compared with individuals ≥ 11 years (78.1%; 95% *CI*: 71.4–84.8) (*P* = 0.02) (Table 4). Rural schoolchildren had a higher prevalence of any STH infection compared with urban schoolchildren (93.4%; 95% *CI*: 86.0–100 vs 79.2%; 95% *CI*: 73.8–84.6; *P* = 0.003) (Table 4). Children in rural schools had higher prevalence of hookworm (39.6%; 95% *CI*: 30.1–49.0) than children in urban schools (26.0%; 95% *CI*: 19.1–32.9; *P* = 0.02) and a higher prevalence of *T. trichiura* (86.8%; 95% *CI*: 78.0–95.6) than children in urban schools (68.2%; 95% *CI*: 61.8–74.6; *P* = 0.001). There was no significant difference in *A. lumbricoides* prevalence between urban and rural schools (*P* = 0.47) (Table 4).

**Table 4:** Prevalence of soil transmitted helminths by gender, age and urban/rural location

	*A. lumbricoides*		*T. trichiura*		Hookworm		Any STH		All 3 STH	
	*n*	Prevalence (95% *CI*)	*n*	Prevalence (95% *CI*)	*n*	Prevalence (95% *CI*)	*n*	Prevalence (95% *CI*)	*n*	Prevalence (95% *CI*)
**KK and qPCR combined**										
Total infections	156	59.1% (53.1–65.1)	197	74.6% (69.3–79.9)	81	30.7% (25.1–36.3)	222	84.1% (79.7–88.5)	64	24.2% (19.0–29.5)
Gender										
Male	78	62.4% (53.7–71.1)	95	76.0% (68.3–83.7)	50	40.0% (32.0–48.0)**	104	83.2% (76.7–89.7)	43	34.4% (27.0–41.8)***
Female	78	56.1% (47.9–64.3)	102	73.4% (66.1–80.7)	31	22.3% (14.7–29.9)	118	84.9% (78.8–91.0)	21	15.1% (8.1–22.1)
Age in years										
Under 11	94	62.7% (54.8–70.6)	117	78.0% (71.0–85.0)	46	30.7% (23.2–38.1)	133	88.7% (82.8–94.5)*	36	24.0% (17.1–30.9)
11 or older	62	54.4% (45.3–63.5)	80	70.2% (62.2–78.2)	35	30.7% (22.2–39.2)	89	78.1% (71.4–84.8)	28	24.6% (16.6–32.5)
School location										
Urban	105	60.7% (53.3–68.1)	118	68.2% (61.8–74.6) **	45	26.0% (19.1–32.9)*	137	79.2% (73.8–84.6)**	38	22.0% (15.5–28.4)
Rural	51	56.0% (45.9–66.2)	79	86.8% (78.0–95.6)	36	39.6% (30.1–49.0)	85	93.4% (86.0–100)	26	28.6% (19.7–37.4)
**qPCR**										
Total infections	145	53.4% (47.4–59.5)	177	67.1% (61.3–72.8)	81	30.7% (25.1–36.3)	208	78.8% (73.8–83.8)	63	23.9% (18.7–29.0)
Gender										
Male	72	57.6% (48.8–66.4)	85	68.0% (59.7–76.3)	50	40.0% (32.0–48.0)**	97	77.6% (70.4–84.8)	42	33.6% (26.2–41.0)***
Female	73	52.5% (44.2–60.8)	92	66.2% (58.3–74.1)	31	22.3% (14.7–29.9)	111	79.9% (73.0–86.7)	21	15.1% (8.1–22.1)
Age in years										
Under 11	86	57.3% (49.3–65.4)	100	66.7% (59.1–74.3)	46	30.7% (23.2–38.1)	123	82.0% (75.4–88.6)	35	23.3% (16.5–30.2)
11 or older	59	51.8% (42.6–61.0)	77	67.5% (58.8–76.2)	35	30.7% (22.2–39.2)	85	74.6% (67.0–82.1)	28	24.6% (16.7–32.5)
School location										
Urban	100	57.8% (50.4–65.3)	106	61.3% (54.3–68.2) **	45	26.0% (19.1–32.9)*	130	75.1% (69.0–81.2)*	37	21.4% (15.0–27.8)
Rural	45	49.5% (39.2–59.7)	71	78.0% (68.4–87.6)	36	39.6% (30.1–49.0)	78	85.7% (77.3–94.1)	26	28.6% (19.8–37.4)
**KK only**										
Total infections	41	15.5% (11.1–19.9)	69	26.1% (20.8–31.5)	0	0%	88	33.3% (27.6–39.1)	0	0%
Gender			35				46			
Male	19	15.2% (8.8–21.6)	34	28.0% (20.2–35.8)			42	36.8% (28.5–45.1)		
Female	22	15.8% (9.8–21.9)	69	24.5% (17.1–31.8)			88	30.2% (22.3–38.1)		
Age in years										
Under 11	26	17.3% (11.5–23.2)	44	29.3% (22.3–36.4)			53	35.3% (27.7–42.9)		
11 or older	15	13.2% (6.5–19.9)	25	21.9% (13.8–30.0)			35	30.7% (22.0–39.4)		
School location										
Urban	22	12.7% (7.3–18.1)	46	26.6% (20.0–33.2)			54	31.2% (24.1–38.3)		
Rural	19	20.9% (13.4–28.3)	23	25.3% (16.2–34.4)			34	37.4% (27.6–47.1)		

*n*: Number of positive cases, 95% *CI* 95% confidence interval, *Any STH* Infection with any of the three parasites (*A. lumbricoides*, *T. trichiura*, or hookworm), *All 3 STH* Infection with all of the three parasites, *KK* Kato-Katz method; Chi-square statistics; significance: *P < 0.05; **P < 0.01; ***P < 0.001

### Infection intensity

The AMEPG and GMEPG values for *A. lumbricoides* and *T. trichiura* obtained by the KK procedure, stratified by infection intensity category as defined by the WHO [20] are shown in Table 5. The majority of infected schoolchildren had low-intensity infections (88.4% with *T. trichiura;* 51.2% with *A. lumbricoides)*. Among 88 infections with any STH (that is either *A. lumbricoides* or *T. trichiura*) by the KK, 23 were moderate-intensity infections (26.1%) and 4 were high-intensity infections (4.6%). Thus, out of all 264 study participants, the proportion of schoolchildren infected with any STH with moderate-to heavy-intensity infections was 10.2% (*n* = 27).

**Table 5:** Soil transmitted helminths infection intensity determined by the Kato-Katz method

	*A. lumbricoides*				*T. trichiura*			
	*n*	% in each category (95% *CI*)	AMEPG (95% *CI*)	GMEPG (95% *CI*)	*n*	% in each category (95% *CI*)	AMEPG (95% *CI*)	GMEPG (95% *CI*)
Light intensity infections	21	51.2 (35.3–67.2)	881.9 (0–2,582.0)	235.5 (129.7–427.6)	61	88.4 (80.7–96.2)	151.7 (107.9–195.6)	86.3 (64.7–115.1)
Moderate intensity infections	16	39.0 (23.4–54.6)	18,703.5 (16,755.8–20,651.2)	14,421.2 (7,294.6–28,575.9)	8	11.6 (3.9–19.3)	2142.0 (2020.9–2263.1)	1958.8 (885.1–4335.1)
Heavy intensity infections	4	9.8 (0.3–19.2)	77,610.0 (73,714.6–81,505.4)	75,335.6 (19,186.7–295,120.9)	0	N/A	N/A	N/A
Total positive	41				69			

*n* Number of positive cases, *95% CI* 95% confidence interval, *AMEPG* Arithmetic mean of eggs per gram of faeces in infected children, *GMEPG* Geometric mean of eggs per gram of faeces in infected children, *N/A* Not applicable

## Discussion

This is the first multiplex molecular epidemiological study to examine the prevalence of four STH species, *A. lumbricoides*, *T. trichiura*, *Ancylostoma* sp*.* and *N. americanus,* in Myanmar. The results indicate that, despite the decade-long, bi-annual targeted deworming programme among pre-SAC (aged 2–4 years) and SAC (fourth grade and below, aged 5–9 years) in Myanmar, STH infections remain highly prevalent among schoolchildren in at least one sentinel site selected for monitoring of the targeted school deworming programme [17].

### STH infection prevalence and intensity of infection

Overall, polyparasitism (determined by qPCR) was also high with 63.5% of infected individuals having two or more infections, and 30.3% of infected individuals having triple infections (Fig. 3).

### *Ascaris lumbricoides* and *Trichuris trichiura*

Results of both KK microscopy and qPCR analysis indicated there was no reduction in infections of *A. lumbricoides* or *T. trichiura* in schoolchildren and, indeed, showed a significantly higher prevalence than previously reported in a MoHS monitoring survey conducted in the same township (Phyu) in 2012 [19]. The 2012 survey, which used KK alone, determined prevalences of 5.8%, 18.6%, and 0.3% for *A. lumbricoides, T. trichiura*, and hookworm respectively [19], much lower than the prevalence values reported here (Table 2). This is not unexpected as qPCR is recognised as being considerably more sensitive than the KK procedure and would thus be expected to identify more infected individuals. However, analysis by KK alone determined prevalences for *A. lumbricoides* and *T. trichiura* of 15.5% and 26.1%, respectively, and these were significantly higher than reported in the earlier MoHS survey utilising the same microscopy-based procedure [19].

Despite its lower sensitivity compared with the qPCR, just over 50% of infections determined using the KK method were light-intensity infections (Table 5). The remainder were classified as moderate-intensity (39.0% of samples) or heavy-intensity infections (9.8% of samples) (Table 5). Although the results obtained by qPCR and KK indicated a very high prevalence overall in schoolchildren with *T. trichiura* (74.6%), no heavy-intensity infection were identified using the KK procedure, and only 11.6% of infected individuals had moderate-intensity infections. These proportions for both *A. lumbricoides* and *T. trichiura* are likely to be underestimates as, for example, unfertilised eggs of *A. lumbricoides* are often missed by the KK [41]. Nevertheless, the proportion of individuals infected with either *A. lumbricoides* or *T. trichiura* in moderate- to heavy-intensity infections (determined by KK) in our study was 10.2%, which is comparable to 10.8% pre-control-programme prevalence of any STH infection of moderate- to heavy-intensity, reported in 2002 in the same study area [19]. This pattern of infection intensity over a period of 14 years indicates there is a long road ahead in order to achieve the WHO-recommended level for STH elimination as a public health problem; that is below 1% prevalence for moderate-intensity and heavy-intensity STH infections [42].

### Hookworm

The 2012 MoHS survey recorded a prevalence of 0.4% for hookworm by the KK procedure [19]. Although we found no positive samples by KK, the hookworm prevalence determined by qPCR was 30.7%, with the majority positive for *Ancylostoma* sp. (Table 2). The low hookworm prevalence recorded by KK is likely due to the rapid egg lysis that occurs post-defecation [41, 43]. To minimise this, stool samples need to be prepared as soon as possible after defecation and slides read within a few hours of preparation [41, 43]. Accordingly, the hookworm results obtained by the KK in this study and from the 2012 national survey [19] are likely underestimated due to egg lysis. *A. ceylanicum*, a zoonotic hookworm of dogs, has recently been identified in Myanmar [44]. The presence of this hookworm species complicates control efforts and will require drug treatment of dogs to help control transmission to humans.

The findings presented here based on prevalence alone underscore the need to up-scale the STH control programme in Myanmar, and again highlight the poor sensitivity of the KK method. The KK stool examinations undertaken in the current study were undertaken with the existing local capacity and limited resources, reflecting the usual scenario in the monitoring of STH control programmes in low-income countries, and reflecting underestimated prevalence/intensity levels in current national surveillance efforts. Accordingly, in addition to KK, which is currently the most-commonly used procedure for monitoring purposes in resource-poor settings [45], more sensitive diagnostic methods, with minimal interference due to other factors such as egg lysis, should be integrated into the monitoring of STH control programmes [44].

### qPCR vs KK

Prevalence determined by the KK procedure was much lower than by the qPCR analysis, a feature of other studies on STH [34, 35, 46, 47]. This reinforces the inadequacy of monitoring STH control programmes using KK alone, particularly in areas with low intensity infections—such as in locations where STH chemotherapy is undertaken. Only 11 stool samples found positive by KK for *A. lumbricoides* were qPCR-negative, whereas 115 samples found positive by qPCR were KK-negative. Similarly, for *T. trichiura* 20 KK-positives were qPCR-negative with 128 KK-negatives positive by qPCR; in regards to hookworm*,* 81 KK-negatives were shown positive by qPCR, possibly due to egg lysis having occurred before KK slides were prepared and read.

Missed infections by qPCR may be due to lack of eggs in the 200 mg stool samples used for DNA extraction or inefficiencies in the DNA extraction procedure – particularly for *T. trichiura* which requires lysis of eggs for release of DNA and subsequent successful amplification by qPCR. The presence of inhibitors in stool is also a factor to consider and will vary from individual to individual. For this reason extracted DNA was diluted 1:5 in water before being added to the PCR reaction as this also reduces the effect of any potential inhibitors and allows the reaction to take place optimally.

The high costs of DNA isolation and qPCR should be taken into consideration in large-scale surveys in low-income countries, where the majority of STH infections occur. We recommend qPCR be used to supplement less expensive approaches (such as faecal floatation, which is more sensitive than the KK procedure [48]) in initial mapping surveys to inform STH control programmes, and in subsequent monitoring when the STH prevalence determined by the KK is shown to be very low.

In addition to PCR methods there are isothermal reaction-based tests that occur at a single temperature which could also be used in resource poor countries as they do not require a thermocycler. Loop mediated isothermal amplification occurs at 65℃ and thus requires a heat block [49] while recombinase polymorphism amplification occurs at 37 ℃ or room temperature [50]. DNA extraction is often the most expensive component of molecular diagnostics assays, and is often a limiting factor precluding their use under field conditions. Nevertheless, there have been several recent advances in the area of crude DNA extraction; these may reduce costs and simplify the procedure for future field work, thereby allowing molecular diagnostics to be more accessible in remote areas where STH are endemic [51, 52].

### The Myanmar National STH control programme, MDA and reinfection

National MDA coverage for 2015 was 95.5% in pre-SAC and 99.2% in SAC [18], a feature which makes the persisting infections after a decade of applying targeted deworming in this Myanmar school setting even more perplexing. At the time of our study, the School and Youth Health Programme in Myanmar targeted only pre-SAC and younger SAC (in the fourth grade and lower) for deworming. Our findings informed the Myanmar National STH control programme in expanding the deworming coverage. As a result, the targeted deworming programme was extended to the entire SAC population (including the fifth grade and above) during the 2017–2018 academic year. Further scale up of the deworming programme should be extended to the entire Myanmar community including adults in endemic areas as adults can still contribute to environmental contamination with helminth eggs and larvae, leading to continued transmission. Similarly, while MDA is effective in treating infected individuals, it cannot prevent re-infection. Re-infection can be prevented by increasing health infrastructure, such as the provision of toilets, access to clean water, and health education, which can increase knowledge and change practice leading to reduced re-infection rates [53, 54].

The finding of the presence of the zoonotic hookworm, *A. ceylanicum,* in another study [44], adds another dimension to the control scenario as dogs can also contribute to ongoing transmission and re-infection. In addition to *A. ceylanicum* there are zoonotic species of *Trichuris* and *Ascaris* (*T. vulpix, T. suis,* and *A. suum*) [55] in canines and pigs which may also contribute to human infection, although these have not been investigated or reported in Myanmar to date.

### Case finding and monitoring

Apart from the 2002 and 2012 MOHS monitoring surveys, which only occurred in one township in each of the four ecological areas [19], no other country-wide assessments to determine the areas endemic for STH in Myanmar have been reported. Given that there are 330 townships in the country, surveys covering more extensive geographical areas are needed to estimate the true extent and distribution of STH infections in Myanmar. STH prevalence determined by microscopy was likely underestimated previously in Myanmar, and we recommend that STH mapping of additional sentinel sites using molecular epidemiological approaches be undertaken initially. Savings in manpower and overhead costs could be made if future community monitoring surveys for STH are conducted in conjunction with transmission assessment surveys for lymphatic filariasis which is widely endemic and another major public health problem in Myanmar [56]. These survey activities will require commitment from the government as a key part of health system strengthening with a strong sense of ownership, and planning for and provision of sufficient resources to invest in the health, growth and wellbeing of Myanmar children, who are the future of the country. The government, non-governmental organisations and academic institutions involved in neglected tropical diseases research should, rather than working in isolation, collaborate and unite to undertake a concerted and effective STH control and monitoring programme in Myanmar.

### Study limitations

In our study, although school selection was randomised, it was conducted in one township on fifth grade students only, resulting in a relatively small but, nevertheless, informative sample size.

The qPCR methodology to determine precise infection intensity is still evolving, and it is usually estimated from Ct scores: the lower the Ct score, the higher the infection intensity [35]. Intensity (eggs per gram of faeces) for *A. lumbricoides* and hookworm was estimated in a previous study using a formula obtained using egg seeding experiments [32] and this study reported that qPCR was more sensitive than KK in determining infection intensity. Some assumptions are made when using qPCR to estimate infection intensity in that: 1) seeding negative stool samples with the correct number of eggs is achieved; 2) DNA is extracted from all eggs present; and 3) DNA is amplified with maximum efficiency and that any potential inhibitors present in human faeces do not affect the qPCR assay. Although some of these issues can be overcome, the use of qPCR to determine infection intensity provides an estimate only. Consequently, the extrapolation of Ct scores into infection intensities should be interpreted with some caution until further validation has been undertaken.

## Conclusions

The results presented here, particularly those obtained by qPCR diagnosis, help contribute to an improved understanding of the current epidemiological picture of STH endemicity in Myanmar. Our findings have informed evaluation of the existing targeted national deworming programme as well as providing the requisite information to plan an integrated STH control and monitoring programme. We found that 84% of fifth grade students were infected with at least one STH species. Polyparasitism was also high with 63.5% harbouring two or more STH species. This is despite a decade of targeted deworming and our results, presented to the MOHS, prompted the expansion of the STH control programme in Myanmar to include older schoolchildren as of 2017. Ultimately deworming should be expanded to the whole community. Currently the national deworming programme is monitored by deworming coverage only [19]. Parasite detection by the KK procedure has occurred only once (in 2012), in one township only from each of the ecological areas, on a total of 1,000 SAC. Additional methods of surveillance should be employed in future and the current programme monitoring may also need to be expanded into new areas. Nationwide surveys would identify locations with potential for STH transmission and infection, which could then be subjected to case finding, control efforts, and ongoing monitoring. Finally, more sensitive parasite detection techniques should be employed particularly in low STH infection intensity areas.

## Data Availability

All required data is included in this manuscript as tables or figures.

## Abbreviations

AMEPG: Arithmetic mean eggs per gram of faeces
EPG: Eggs per gram of faeces
GMEPG: Geometric mean eggs per gram of faeces
KK: Kato-Katz
MoHS: Ministry of Health and Sports
PCR: Polymerase chain reaction
qPCR: Real time PCR
QIMRB: QIMR Berghofer Medical Research Institute
STH: Soil transmitted helminths
SAC: School aged children
WHO: World Health Organization
